# Cost-effectiveness of DTG+ABC/3TC versus EFV/TDF/FTC for first-line treatment of HIV-1 in the United States

**DOI:** 10.7448/IAS.17.4.19605

**Published:** 2014-11-02

**Authors:** Siyang Peng, Ali Tafazzoli, Emily Dorman, Lisa Rosenblatt, Angelina Villasis-Keever, Sonja Sorensen

**Affiliations:** 1 Evidera, Modeling & Simulation, Bethesda, MD, USA; 2 Health Economics & Outcomes Research, Bristol-Myers Squibb Company, Plainsboro, NJ, USA; 3 HIV Medical Strategy, Bristol-Myers Squibb Company, Plainsboro, NJ, USA

## Abstract

**Introduction:**

Data from the SINGLE trial demonstrated that 88% of treatment-naive HIV-1 patients treated with dolutegravir and abacavir/lamivudine (DTG+ABC/3TC) achieved viral suppression at 48 weeks compared with 81% of patients treated with efavirenz/tenofovir disoproxil fumarate/emtricitabine (EFV/TDF/FTC). It is unclear how this difference in short-term efficacy impacts long-term cost-effectiveness of these regimens. This study sought to evaluate the long-term cost-effectiveness of DTG+ABC/3TC versus EFV/TDF/FTC from a US payer perspective.

**Materials and Methods:**

An individual discrete-event simulation model tracked the disease status and treatment pathway of HIV-1 patients. The model simulated treatment over a lifetime horizon by tracking change in patients’ CD4 count, occurrence of clinical events (opportunistic infections, cancer and cardiovascular events), treatment switch and death. The model included up to four lines of treatment. Baseline patient characteristics, efficacy and safety of DTG+ABC/3TC and EFV/TDF/FTC were informed by data from the SINGLE trial. The efficacy of subsequent lines of treatment, clinical event risks, mortality, cost and utility inputs were based on literature and expert opinion. Outcomes were lifetime medical costs, quality-adjusted life-years (QALYs) (both discounted at 3% per annum) and the incremental cost-effectiveness ratio (ICER).

**Results:**

Compared with EFV/TDF/FTC, DTG+ABC/3TC increased lifetime costs by $58,188 and per-person survival by 0.12 QALYs, resulting in an ICER of $482,717/QALY. In sensitivity analyses testing conservative assumptions about EFV/TDF/FTC's efficacy beyond the trial period, ICERs comparing DTG+ABC/3TC to EFV/TDF/FTC remained high (lowest reported ICER of $365,662/QALY). In a scenario in which the price of EFV/TDF/FTC was reduced by 10% to reflect the potential for price reduction as EFV goes off patent, DTG+ABC/3TC's ICER compared to EFV/TDF/FTC was $600,916/QALY. When DTG+ABC/3TC's price was reduced by 10%, the resulting ICER comparing DTG+ABC/3TC to EFV/TDF/FTC was $302,171/QALY.

**Conclusions:**

Compared with EFV/TDF/FTC, DTG+ABC/3TC resulted in substantially higher cost, slightly better QALY over lifetime, and ICERs far exceeding standard cost-effectiveness thresholds, indicating that the incremental benefit in efficacy associated with DTG+ABC/3TC may not be worth the incremental increase in costs.

